# AVPdb: a database of experimentally validated antiviral peptides targeting medically important viruses

**DOI:** 10.1093/nar/gkt1191

**Published:** 2013-11-26

**Authors:** Abid Qureshi, Nishant Thakur, Himani Tandon, Manoj Kumar

**Affiliations:** Bioinformatics Centre, Institute of Microbial Technology, Council of Scientific and Industrial Research, Sector 39-A, Chandigarh-160036, India

## Abstract

Antiviral peptides (AVPs) have exhibited huge potential in inhibiting viruses by targeting various stages of their life cycle. Therefore, we have developed AVPdb, available online at http://crdd.osdd.net/servers/avpdb, to provide a dedicated resource of experimentally verified AVPs targeting over 60 medically important viruses including Influenza, HCV, HSV, RSV, HBV, DENV, SARS, etc. However, we have separately provided HIV inhibiting peptides in ‘HIPdb’. AVPdb contains detailed information of 2683 peptides, including 624 modified peptides experimentally tested for antiviral activity. In modified peptides a chemical moiety is attached for increasing their efficacy and stability. Detailed information include: peptide sequence, length, source, virus targeted, virus family, cell line used, efficacy (qualitative/quantitative), target step/protein, assay used in determining the efficacy and PubMed reference. The database also furnishes physicochemical properties and predicted structure for each peptide. We have provided user-friendly browsing and search facility along with other analysis tools to help the users. Entering of many synthetic peptide-based drugs in various stages of clinical trials reiterate the importance for the AVP resources. AVPdb is anticipated to cater to the needs of scientific community working for the development of antiviral therapeutics.

## INTRODUCTION

Viruses are the causative agents of various dreadful diseases in humans and animals (1,2). For majority of viruses like Hepatitis C virus (HCV), Influenza, Dengue virus (DENV), Severe acute respiratory syndrome (SARS), Herpes simplex virus (HSV), etc., antiviral therapeutics are limited or lacking (3). Moreover, owing to increasing drug resistance, conventional antiviral therapy is continuously challenged these days (4,5). Therefore, scientific efforts are underway to search for novel antivirals (6,7). Antiviral peptides (AVPs) are being regarded as such new promising entities to combat the viral infections.

AVPs are a subset of antimicrobial peptides (AMPs) which act as the first line of defence in many organisms as innate immune response and are the hosts’ defence peptides generated in response to pathogenic disease condition (8–10). AVPs are known to act either directly or by eliciting immune response (11). They usually inhibit directly one or more stages in the life cycle of a virus, viz., entry, attachment, replication, transcription, translation, maturation, release, etc.; thereby exhibiting the antiviral effects (12,13).

One of the earliest reports stating the direct involvement of peptides in inhibiting Herpes simplex virus (HSV) multiplication dates back to 1985 (14). Since then researchers have been extensively working on peptide-based antiviral development. Bultmann *et al.* (15) used FGF4 signal peptide derivatives to inhibit HSV-1 entry and the best performing AVP had a half maximal inhibitory concentration (IC_50_) of 0.7 μM. Budge and Graham (9) used ρ-A derived peptides to inhibit Respiratory syncytial virus (RSV) replication and achieved a maximum IC_50_ of 1.23 μM. Also, a peptide derived from spike (S) protein of SARS-CoV has been proved to be effective against SARS virus entry with an efficacy of 11 μM (16). The peptide ‘FluPep’ inhibits Influenza virus attachment to the cells with an IC_50_ of 0.10 μM (17). Similarly, Xu *et al.* (18) were able to inhibit DENV protease using AVPs with a minimum IC_50_ of 3.3 μM. An AVP named ‘Ctry2459’ has been synthesized, which possesses anti-HCV activity with an EC_50_ of 1.84 μg/ml (19). Therapeutic potential, mode of action and importance of AVPs has been further reviewed (8,10,20).

Peptide-based drugs are advantageous over conventional drugs in having lesser molecular weight, higher efficiency, lower toxicity and minor side effects (21). AVPs are usually derived from natural sources but they can be readily modified by adding chemical groups or non-natural amino acids to further enhance their activity and stability (22). Due to high potential, an estimated 15 peptide-based therapeutics as antimicrobial/immunomodulatory are under clinical trials (23). The first AVP to pass the clinical trials was ‘Enfuvirtide’ (T20), an HIV fusion inhibitor that is being sold under the name of ‘Fuzeon’ (24,25).

Bioinformatics resources are required to accommodate and analyse the enormous data being generated on AVPs. Although a number of resources exist for general antimicrobial peptides like APD2 (26), CAMP (27), DAMPD (28), YADAMP (29), LAMP (30), etc., yet, specific resources on AVPs are lacking. Therefore, to fill this void we have recently developed AVPpred (13) and HIPdb (12). AVPpred is the first AVP prediction algorithm developed using Support Vector Machine (SVM). Whereas HIPdb is a specific database of experimentally validated HIV inhibiting peptides, which is freely available at http://crdd.osdd.net/servers/hipdb. HIPdb harbours information of 981 peptides and 87 modified peptides experimentally tested for HIV inhibiting activity. Besides above, no other resource is available for AVPs. Hence, we developed AVPdb—a comprehensive resource of peptides experimentally validated for their antiviral activities.

## DATA ACQUISITION

Relevant data were retrieved from the PubMed database, a free repository of abstracts and references on biomedical and life sciences. Exhaustive literature search was accomplished by building search queries having combination of many keywords including virus, viral, peptide, inhibit, block, etc. A typical text mining query is given below:

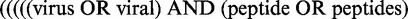


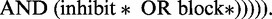



Full text search returned 37 842 articles as on 1 July 2013. In the initial screening, we found that majority of the articles were not furnishing the desired data. This could be due to the fact that the above keywords are quite frequent in the literature. Therefore, we limited our query to the title/abstract fields and retrieved 5000 articles using the advanced search option of PubMed. These articles were manually examined in detail based on their abstracts/full paper to fish out the desired data. Besides, we have also searched these keywords in the PatentLens database and included data from eight relevant patents. Reviews, general methodological and non-English articles were not considered. Besides these, there were number of articles in which information on only predicted peptides or peptide structures or analogues was given were excluded. Also, dendrimeric peptides, complex peptide conjugates and peptide/drug combinations were removed. Similarly, articles that were lacking peptide sequence or experimental efficacy were also not considered. In addition, peptides targeting HIV were also left out of the database, as these data were already published in our recent database, HIPdb (12). Papers that were limited in giving information only on predicted peptides or design, peptide structural studies, peptide analogues, dendrimeric peptides, complex peptide conjugates, peptides used in combination with drugs, emphasis was laid on to articles having experimentally validated peptides and covering all or most of the AVPdb fields. After filtering out the above articles, remaining 263 research articles were finally used to collect 2059 peptides experimentally tested for virus inhibiting activity. Further 624 modified peptides were also extracted and have been provided separately in AVPdb. In our database, complete AVP data of almost all human viruses reported in the literature have been included.

### Database architecture

**Figure 1.**
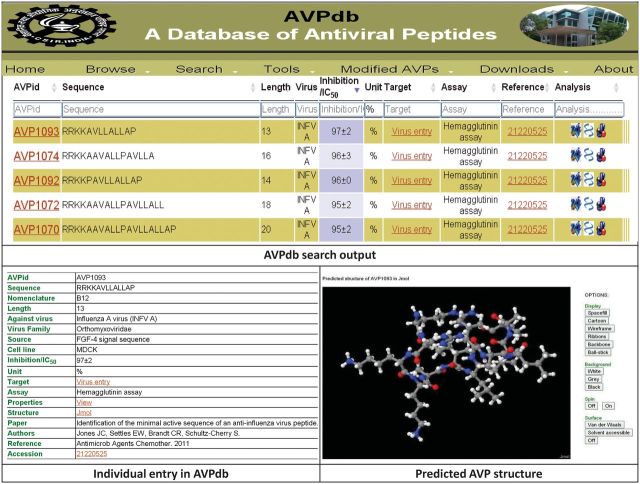
AVPdb overview including search output, individual entry and predicted structure.

AVPdb is a manually curated, open source database of AVPs targeted against diverse viruses of therapeutic importance. The database comes with easy-to-operate browsing as well as searching with sorting and filtering functionalities. AVPdb also provides physicochemical properties and predicted structure of AVPs along with more informative tools for data analysis such as BLAST and MAP as well as links to major peptide resources. Physicochemical properties displayed are charge, polarity, composition, hydrophobicity and secondary structure preference. The values used for calculating these properties were retrieved from the AAindex database. Structures of the peptides were predicted using the PepStr algorithm (31) and PEP-FOLD (32) server. Structures are displayed in Jmol applet. To view the structures, java plugin should be installed in the browser and JavaScript to be enabled. BLAST and MAP tools help in finding the similar peptides reported in the database. Overall database architecture is shown in Figure 1.

AVPdb currently archives the following fields extracted from the literature:

*Sequence*: all peptide sequences are formatted in standard one letter amino acid notation along with their respective string length.
*Nomenclature*: peptide designation from the respective study.
*Virus*: the target virus of the antiviral peptide. Also included are virus abbreviations and taxonomy.
*Source*: the origin of the peptide including the organism name/parent protein.
*UniprotKB ID*: the Uniprot accession number of the source protein (if available).
*Cell line*: cells on which the experiments are performed.
*Inhibition*: the inhibitory activity of the peptide, either qualitative or quantitative as discussed in the respective research article. Different workers have used varying measures of peptide efficacy. These are percentage inhibition, IC_50_, half maximal effective concentration (EC_50_), 90% inhibitory concentration (IC_90_), inhibition/dissociation constants, etc.
*Unit*: unit(s) of quantitative information (e.g. μM, nM, percentage, μg/ml, etc.) provided in the preceding column.
*Target*: the protein/molecular process targeted by the peptide to inhibit the virus.
*Assay*: experimental validation method.
*Reference*: references are given as PubMed IDs with external links pointing to the abstract of the article. Respective author and journal information for each entry are also included in the database.

### Database statistics

**Figure 2.**
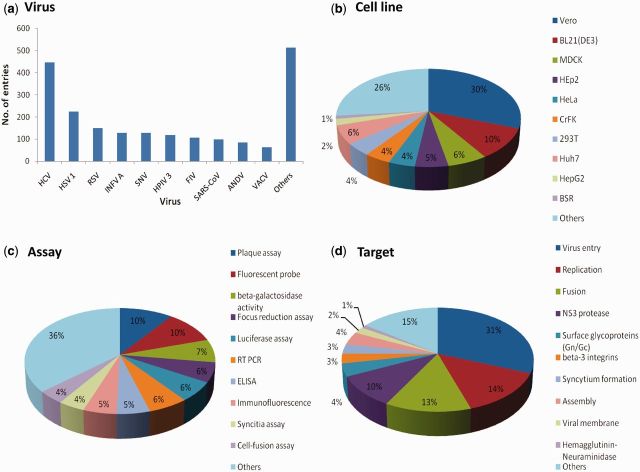
AVPdb statistics of peptides with natural amino acids. (**a**) Viruses, (**b**) Cell lines, (**c**) Assays and (**d**) Targets.

**Figure 3.**
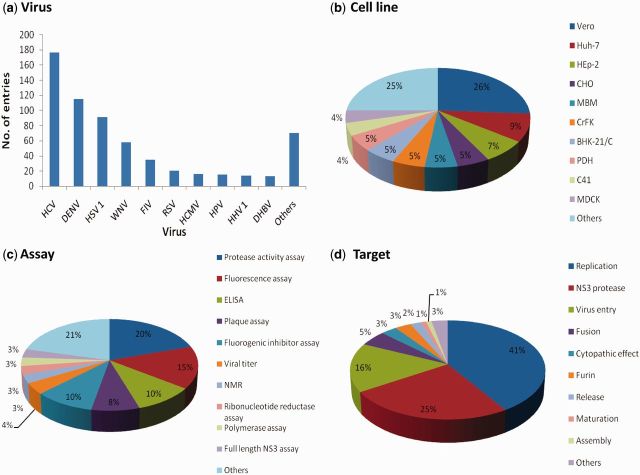
AVPdb statistics of peptides with modified amino acids. (**a**) Viruses, (**b**) Cell lines, (**c**) Assays and (**d**) Targets.

AVPdb is a manually curated, open source database of AVPs targeted against more than 60 viruses. The database comprises experimentally verified 2059 normal peptides spanned over varied efficacies and lengths, reported in the literature and tested on 85 cell lines. About 624 modified peptides having additional chemical groups are presented under modified AVPs. Among the different cell lines it was observed that Vero, BL21 (DE3), MDCK, CrFK, HeLa and Huh7 were mostly used. Also, we found that the peptides were directed against over 40 different targets of which virus entry, replication, fusion, NS3 protease, surface glycoproteins and β-3 integrins were more commonly aimed to inhibit the viruses. From among the different viruses, HCV, HSV 1, Influenza A virus (INFV A), Sin Nombre virus (SNV), Human parainfluenza virus type 3 (HPIV 3), Feline leukaemia virus (FIV), RSV and West Nile virus (WNV) were found to be most commonly targeted. These statistics are depicted in Figure 2. These facts were also separately calculated for modified peptides as shown in Figure 3. Further analysing the overall amino acid composition of the database, it was noticed that some amino acids like Leu, Lys, Ala, Arg and Val were found to be more abundant while some amino acids like His, Met, Trp and Tyr were present less frequently. These results are shown in Supplementary Figure S1. Peptide efficacy statistics for natural as well as modified peptides are presented in Table 1. Also, the top sources of the natural and modified peptides are given in Table 2.

**Table 1.** Efficacy unit statistics of peptides in AVPdb

Sl no.	AVP efficacy unit	No. of AVPs	
		Natural	Modified^a^
1	Percentage inhibition (%)	637	4
2	Half maximal inhibitory concentration (IC_50_)	610	425
3	Half maximal effective concentration (EC_50_)	122	38
4	90% inhibitory concentration (IC_90_)	106	0
5	Dissociation constant (*K*_d_)	19	1
6	Inhibition constant (*K*_i_)	12	147
8	Qualitative (high/medium/low)	649	9

^a^These peptides are comprised of non-natural or chemically modified amino acids.

**Table 2.** Sources of natural and modified AVPs

Sl no.	AVP source^a^	Entries	Modified AVP source^b^	Entries
1	Phage display	313	Synthetic	316
2	HCV polyprotein	199	Combinatorial peptide library	66
3	Synthetic	197	Dengue capsid protein	45
4	HSV-1 B glycoprotein (gB)	144	RSV attachment glycoprotein	20
5	HPIV3 fusion (F) protein	124	HCV NS4A/NS4B cleavage site	16
6	RSV fusion (F) protein	95	HSV DNA polymerase (C-terminal residues)	16
7	FGF-4 signal sequence	84	FIV envelope glycoprotein (gp41)	15
8	SARS-CoV spike protein	76	Halovirs (*Scytalidium* fungus)	14
9	FIV envelope glycoprotein (gE)	66	HSV ribonucleotide reductase subunit 2	14
10	HCV non-structural protein 5A	58	Phage display	14
11	Others	703	Others	88

^a^These peptides are composed of natural amino acids.

^b^These peptides are comprised of non-natural or chemically modified amino acids.

## TOOLS

### (i) AVPdb MAP

The ‘AVPdb MAP’ is a user friendly tool to fetch the perfectly matching peptide available in our database. So, it helps the user to find how many peptides against the user-provided protein sequence are available in our database. The output of this tool displays the AVPid, its source, sequence and its target. Also mentioned is the start position where the match is found in the user-provided sequence.

### (ii) AVPdb BLAST

Additionally, the BLAST allows alignment of a user-provided peptide sequence against all the peptide sequences available in our database. This helps the user to confirm whether a given peptide sequence or similar one has already been reported or not. The output is given in the standard format with the BLAST score and *e*-value. The alignment is shown for the peptides found to be identical or similar in the database. The output can be formatted based on the options provided by user.

### (iii) Physicochemical properties calculator

Various important physicochemical properties such as amino acid composition, hydrophobicity, preference for β-sheets, frequency of α-helix, amino acid charge and polarity can been calculated using AAindex (33). These properties can be calculated for any user-provided peptide sequence by submitting it on the analysis page available under tools column.

### Data retrieval

A user-friendly ‘Browse by’ option allows to explore the data for normal peptides by any of the fields categorized in the database, viz., Virus, Family, Peptide Source, Cell Line, Target and Assay. For modified AVPs also, a separate browse option is provided where the data can be sought by Virus, Modification, Peptide Source, Cell Line, Target and Assay. To specifically retrieve HIV inhibiting peptides, extensive links of HIPdb are provided from AVPdb pages.

AVPdb has been incorporated with four different searches:

- Field Search: here the user can enter the query in the box and can specify any of the 10 fields against which one wishes to search or else keep the default ‘all’ option which will search against all the fields in the database. Besides the option to choose the fields, search type allows to retrieve either an exact match or the match containing the query. The results obtained from this search display 10 fields where first nine contain the experimental data and the last one, ‘Analysis’, has links to BLAST results, physicochemical properties and predicted peptide structure. The results retrieved here can be exported to a comma separated value (.csv) file. The data can be sorted by clicking on the column header and filtering can be done by typing in the boxes given under the headers.
- Advanced Search: this feature helps to build custom search with logical operators like AND/OR using multiple queries.
- Conditional Search: this additional search facility allows to add conditions like >, <, = or LIKE to define the query and also helps building the searches using logical operators in a single query. This search has further two divisions: (a) ‘Flexible varchar’ (variable character) search and (b) Numerical search. The former is suitable for variable text-based search, whereas the latter is optimized for numbers. The result table can be sorted in increasing/decreasing order by clicking the arrows on the table header in any column. Also, one can search for any desired keyword in the output by typing the word in the Filter box.
- Multi-display Search: this option enables the user to choose the fields to be searched as well as the fields to be displayed using checkboxes. This search allows displaying multiple fields and records. The result output can be further filtered by typing the desired keyword in the filter box. Search output can be downloaded as .csv file by clicking on the download button.

### Data submission

As more and more AVPs are being published, the interested workers may submit the desired data into AVPdb via the online submission form provided in the database. Once the information is cross-verified by our team, it will be included in the updates of database.

## IMPLEMENTATION

AVPdb database is implemented using the open source LAMP solution stack on Red Hat Enterprise Linux 5 (IBM SAS ×3800 machine) with MySQL (5.0.51b) and Apache (2.2.17) in back-end and front-end of web interface is implemented with PHP (5.2.14). The database is freely available at http://crdd.osdd.net/servers/avpdb.

## FUTURE DEVELOPMENTS

A vast amount of data regarding AVPs both natural as well as modified is reported every year. To cope with these valuable data, we would like to include more viruses or newly discovered unique peptides to our database as appropriate information becomes available in the scientific literature. Also, a tool to predict the IC_50_ value of virus inhibitory peptides shall be plugged in the database in near future.

## SUPPLEMENTARY DATA

Supplementary Data are available at NAR Online.

## FUNDING

The Council of Scientific and Industrial Research, India (GENESIS-BSC0121); the Department of Biotechnology, Government of India (GAP001). Funding for open access charge: CSIR-Institute of Microbial Technology, Chandigarh, India.

*Conflict of interest statement*. None declared.
